# Superficial ocular vascular changes after orbital decompression in patients with thyroid ophthalmopathy measured by anterior segment OCT angiography; an observational study

**DOI:** 10.1038/s41598-024-64925-5

**Published:** 2024-06-24

**Authors:** Seyed Mohsen Rafizadeh, Mostafa Heidari, Amirhossein Aghajani, Zahra Montazeriani, Pedram Afshar, Sajad Mansourian, Ahmad Masoumi, Mohammad Taher Rajabi

**Affiliations:** 1 Department of Plastic and Reconstructive Surgery, Farabi Eye Hospital, Tehran University of Medical Sciences, Tehran, Iran; 2 Department of Medical Physics and Biomedical Engineering, Tehran University of Medical Science, Tehran, Iran; 3 Eye Research Center, Farabi Eye Hospital, Faculty of Medicine, Tehran University of Medical Sciences, Tehran, Iran; 4 Department of Ophthalmology and Eye Research Center, Farabi Eye Hospital, Tehran University of Medical Sciences, Tehran, 133661635 Iran

**Keywords:** Anterior segment-OCT angiography, Ocular surface, Thyroid eye disease, Eye diseases, Endocrine system and metabolic diseases

## Abstract

Thyroid eye disease (TED) is a common ophthalmologic manifestation of thyroid dysfunction. Despite various imaging techniques available, there hasn't been a widely adopted method for assessing the anterior segment vasculature in TED patients. Our study aimed to evaluate alterations in ocular surface circulation following orbital decompression surgery in TED patients and investigate factors influencing these changes. Using anterior segment optical coherence tomography-angiography (AS-OCTA), we measured ocular surface vascularity features, including vessel density (VD), vessel diameter index (VDI), and vessel length density (VLD), both before and after decompression surgery, alongside standard ophthalmic examinations. Our AS-OCTA analysis revealed a significant decrease in most of the temporal vasculature measurements six weeks post-surgery (p < 0.05). However, differences in the nasal region were not statistically significant. These findings indicate notable changes in ocular surface circulation following orbital decompression in TED patients, which may have implications for intraocular pressure (IOP) control and ocular surface symptoms management. AS-OCTA holds promise as a tool for evaluating the effectiveness of decompression surgery and assessing the need for further interventions.

## Introduction

Optical coherence tomography angiography (OCTA) is a non-invasive imaging method that swiftly captures images of all retinal layers while simultaneously visualizing retinal vessel blood flow^1,2^. Alternative techniques such as slit lamp photography, fluorescein angiography, and indocyanine green angiography exist, but they have limitations due to the use of dyes^3–6^. In contrast, OCTA requires no eye contact and aids significantly in disease diagnosis, making it suitable for examining the anterior eye segment. Anterior segment OCT angiography has been used to map the vasculature of the cornea, conjunctiva, sclera, and iris, as well as to visualize neovascularization and associated hyperemia and ischemia^7^. Recent studies have also used OCTA to evaluate ocular surface vasculature changes post-pterygium surgery and keratoplasty^4,8,9^. Moreover, AS-OCTA can detect the increase in episcleral venous congestion observed in patients with carotid-cavernous fistulas, a condition typically imaged using more invasive methods^10^. Interestingly, OCTA of the retina can simultaneously assess peripapillary vessels to demonstrate early glaucomatous damage in carotid-cavernous fistulas.

Thyroid eye disease (TED) is an orbital condition frequently associated with thyroid dysfunction. While it affects women 2.5–6 times more often than men, men tend to experience more severe forms and are usually older. TED typically begins between the ages of 30 and 50, with its severity often escalating after age 50^11^. The disease presents a wide range of clinical signs and symptoms, with inflammatory factors often initiating the process^11^. These symptoms may include peri-orbital edema, conjunctival hyperemia, chemosis, and superior limbic keratoconjunctivitis. Peri-orbital edema, in particular, can result in eyelid fat prolapse, venous drainage disorder, and retroseptal infiltration^11^.

Recent research suggests that the circulation of ocular structures changes in patients with thyroid eye disease (TED). Studies examining the superior ophthalmic vein have shown decreased flow in TED patients^12^. Efficient orbital decompression surgery has been found to increase blood flow velocity in the superior ophthalmic vein (SOV), leading to a reduction in episcleral venous pressure and episcleral venous congestion^13^. As exophthalmos decreases, arterial resistance also decreases^14^. Additionally, TED patients often exhibit increases in choroidal thickness and choroidal vasculature, likely due to enlarged extraocular muscles and other orbital tissues as well as orbital congestion^15^.

Despite vascular changes in the ocular surface being common symptoms of thyroid eye disease (TED), assessments of ocular surface vasculature are less explored. Existing scoring systems for TED activity primarily focus on conjunctival and scleral redness (clinical activity score [CAS] and VISA). Given the impact of efficient orbital decompression on superior ophthalmic vein (SOV) blood flow and episcleral venous pressure, evaluating changes in ocular surface circulation could validate the success of decompression surgery. However, imaging methods for ocular surface assessment are less frequently employed^16^. Considering the widespread use of qualitative assessments of ocular surface vasculature changes in TED diagnosis and the necessity for surgery in these patients, our aim was to investigate conjunctival and scleral circulation using OCT-A in TED patients before and after decompression surgery, to conduct a quantitative assessment of ocular surface vasculature.

## Methods

This study adhered to the Declaration of Helsinki and received approval from the Ethics Committee of Farabi Eye Hospital. All participants provided written informed consent before enrollment.

### Study participants

In this prospective observational study, we evaluated patients clinically diagnosed with thyroid eye disease (TED) who visited the orbit and oculoplastic clinic at Farabi Eye Hospital in Tehran, Iran. The study spanned 14 months, from January 2023 to February 2024. Patients with confirmed TED who planned to undergo decompression surgery were consecutively enrolled. Demographic data, ophthalmic examinations, and vascular features were documented one week before and six weeks after decompression surgery. Exclusion criteria included the inability to undergo OCT and OCT-A imaging, the presence of concurrent ocular surface diseases (e.g., glaucoma, diabetic retinopathy, and any optic neuropathy unrelated to TED), active thyroid eye disease, prior orbital decompression, strabismus, or eyelid surgery, and uncontrolled thyroid function tests.

Demographic data and past medical history, including smoking history, thyroidectomy history, and treatment with radioactive iodine, were documented through interviews. All enrolled patients underwent comprehensive ophthalmic examinations, which included best corrected visual acuity (BCVA), Hertel exophthalmometry, margin-to-reflex distance (MRD) measurements of 1 and 2, and intraocular pressure (IOP) measurements.

Disease activity was evaluated using the clinical activity score (CAS), which assesses spontaneous retrobulbar pain, pain during vertical eye movement, eyelid erythema, eyelid edema, conjunctival injection, conjunctival chemosis, and caruncle inflammation. Each of these criteria was scored. Patients with CAS scores ≥ 3 were classified as having active TED and were excluded from the study.

### Surgical technique

To decompress the medial orbital wall, a medial transcaruncular orbitotomy was performed under general anesthesia, with a blepharostat in place. The procedure began with a conjunctival incision in the medial part, extending under the caruncle using Westcott scissors. Dissection was then carried out to the periorbita level. The medial orbital wall was exposed by incising the exposed periorbita. Subsequently, bone was removed posterior to the posterior lacrimal crest and inferior to the ethmoidal arteries. Finally, the conjunctiva was repaired using an 8/0 Vicryl suture.

If inferior wall decompression was needed due to severe proptosis, requiring double-wall decompression, it was performed during the same session. The periosteum was incised, and the orbital rim was exposed. The medial portion of the orbital floor was removed medial to the infraorbital nerve canal and extended posteriorly to the posterior wall of the maxillary sinus. Bone was preserved one centimeter behind the inferior orbital rim and at the junction of the lower and medial walls of the orbit. Finally, the conjunctiva in the inferior fornix was repaired using an 8/0 Vicryl suture.

### Anterior segment optical coherence tomography angiography

All study participants underwent AS-OCTA imaging at the designated imaging site. Experienced photographers conducted the imaging sessions using the AngioVue OCTA system manufactured by Optovue, CA, USA. The total scan acquisition time for AngioVue OCTA was less than 3 s. The device operated at a central wavelength of 840 nm with a beam width of 22 µm. It achieved a scanning speed of 70,000 A-scans per second and provided an optical axial resolution of approximately 5 µm. The device acquired 304 × 304 A-scans, capturing two consecutive B-scans at each position to differentiate between static tissues and structures with high signal fluctuations. Blood flow was detected by combining the two images at the same location using proprietary angiography algorithms and motion correction techniques. The split spectrum amplitude decorrelation angiography (SSADA) algorithm in AngioVue enhances the signal-to-noise ratio and improves flow detection.

### Image acquisition and segmentation

We employed the Angioretina mode of the AngioVue OCTA system to capture the vasculature of the anterior segment of the eye. Separate images were captured for the temporal and nasal halves of each eye. Vascular parameters of each scanned image, including vessel density (VD), vessel length density (VLD), and vessel diameter index (VDI), were assessed at two distinct depths:

- **Superficial**: From the conjunctival epithelium to a depth of 200 µm.
- **Deep**: From a depth of 200 µm to 1000 µm, indicating the intrascleral layer.

The conjunctival area within each image was manually identified by aligning it with the inner limiting membrane as indicated by the device. Each image was further subdivided into temporal and nasal halves for detailed analysis.

### Vascular indices in anterior segment OCT angiography (OCT-A)

In anterior segment OCT-A, the following indices are used to analyze the vascular density and blood flow characteristics of the eye's anterior segment:

- **Vessel Density (VD)**: VD represents the percentage of the area occupied by blood vessels within a specific region of interest (ROI). It provides information about the overall vessel density in that area.
- **Vessel Length Density (VLD)**: VLD measures the total length of blood vessels per unit area in a specific ROI, giving information about the length of vessels within a given region.
- **Vessel Diameter Index (VDI)**: VDI calculates the average diameter or caliber of blood vessels within a specific ROI, providing information about vessel caliber or thickness.

These indices were calculated using specialized software that analyzes OCT-A images and extracts the relevant vascular parameters.

### Statistical analysis

Descriptive statistics, including mean and standard deviation, were calculated for the study variables. We compared the means of the parameters before and after the operation. Given the inclusion of data from both eyes of the same individuals, we used Generalized Estimating Equations (GEE) analysis to account for the correlation between repeated measures on the same subjects before and after surgery. So, we model the change in predictor variables as a function of time (pre- and post-decompression). The coefficient was reported and results were considered significant if p-values were less than 0.05.

We constructed a generalized linear model in which vascular features were the dependent variables, while age, sex, demographic data, and ophthalmic data (including LogMAR BCVA, MRD1, MRD2, CAS, and IOP) were considered independent variables. Missing data were excluded from the analysis. To determine the effect of each independent variable on vascular features, we used multivariable analysis. Statistical significance was defined as a p-value less than 0.05. All statistical analyses were performed using IBM SPSS Statistics software, version 27 (IBM Corp, New York, USA).

### Sample size

The primary outcomes of this study were changes in vessel parameters, including density, length density, and diameter indexes. We calculated the maximum required sample size for all these parameters based on the observed standard deviation of the changes and aimed to detect at least a 10% change in the parameters from baseline. The parameter that required the largest sample size was VDI nas/sup. To detect a 5% change in this parameter with 90% power, a minimum of 31 eyes would have been needed.

### Ethics approval and consent to participate

The study adhered to the Declaration of Helsinki. Ethics committee approval was obtained from the Tehran University of Medical Sciences. All study participants provided written informed consent prior to enrollment.

## Results

Thirty-one eyes from 22 patients with thyroid eye disease (TED) were included in this study, with a sex distribution of 64% female (14 patients). The mean (± SD) age of the patients was 45.36 (± 10.35) years. The demographic data of the patients are shown in Table 1.

**Table 1:** Participants demographics and clinical features of TED patients.

Demographics	Case	Percent
Female	14	64
Age, mean year (± SD)	45.36 (± 10.35)	–
Hx of smoking	6	36
Hx of thyroidectomy	3	14
Hx of iodotherapy	11	50

*SD* standard deviation, *Hx* history.

The ocular surface vasculature in both deep and superficial layers was measured before decompression surgery. The results of these measurements, along with the results of the preoperative ocular examinations, are presented in Table 2. No significant differences were observed between men and women regarding AS-OCTA parameters. However, nasal-superficial vessel density (VD), deep-temporal VD, nasal-superficial vessel length density (VLD), deep-nasal VLD, and deep-temporal VLD were found to be greater in smokers compared to nonsmokers.

**Table 2:** Comparison of ophthalmic examination between subgroups.

Characteristics	Mean ± SD			Mean ± SD			Mean ± SD		
	Female	Male	p-value	Non-smokers	smokers	P-value	No Hx of thyroidectomy	Hx of thyroidectomy	P-value
VA (logMAR)_pre	0.07 ± 0.12	0.05 ± 0.12	0.93	0.08 ± 0.14	0.01 ± 0.02	0.032	0.07 ± 0.13	0 ± 0	0.14
Exophthalmos_Pre	24 ± 3.8	26.5 ± 2	0.015	24.2 ± 3.5	27 ± 2.5	0.18	24.9 ± 3.6	24.7 ± 0.6	0.01
MRD1_Pre	6 ± 2.1	6.9 ± 3.6	0.034	6.1 ± 2	6.8 ± 4.2	< 0.001	6.4 ± 2.6	5.3 ± 3.5	0.75
MRD2_pre	6.6 ± 1.2	6.8 ± 1.8	0.361	6.2 ± 1	7.9 ± 1.6	0.31	6.7 ± 1.4	6.5 ± 1.3	0.82
IOP_pre	16.5 ± 3.5	17.6 ± 2.2	0.081	16.8 ± 3.1	17.3 ± 3.2	0.64	16.8 ± 3.2	17.7 ± 0.6	< 0.01
CAS_Pre	0.7 ± 0.92	1.36 ± 0.81	0.867	0.91 ± 0.95	1 ± 0.93	0.50	0.96 ± 0.92	0.67 ± 1.15	0.44
Vessel density-nasal superficial	0.37 ± 0.07	0.42 ± 0.08	0.64	0.38 ± 0.05	0.41 ± 0.12	< 0.001	0.38 ± 0.07	0.41 ± 0.11	0.44
Vessel density-nasal deep	0.31 ± 0.07	0.37 ± 0.09	0,70	0.32 ± 0.06	0.37 ± 0.12	0.06	0.34 ± 0.07	0.29 ± 0.16	0.01
Vessel density-temporal superficial	0.4 ± 0.08	0.41 ± 0.07	0.90	0.4 ± 0.08	0.4 ± 0.08	0.52	0.4 ± 0.08	0.4 ± 0.06	0.52
Vessel density-temporal deep	0.34 ± 0.11	0.38 ± 0.1	0.85	0.35 ± 0.1	0.36 ± 0.14	0.03	0.36 ± 0.11	0.34 ± 0.11	0.56
Vessel length density-nasal superficial	0.18 ± 0.03	0.21 ± 0.04	0.42	0.19 ± 0.02	0.19 ± 0.06	< 0.001	0.19 ± 0.03	0.21 ± 0.07	0.04
Vessel length density-nasal deep	0.16 ± 0.03	0.2 ± 0.05	0.13	0.17 ± 0.03	0.19 ± 0.07	0.01	0.18 ± 0.03	0.16 ± 0.11	< 0.001
Vessel length density-temporal superficial	0.2 ± 0.03	0.21 ± 0.03	0.83	0.2 ± 0.03	0.21 ± 0.04	0.31	0.2 ± 0.03	0.21 ± 0.03	0.83
Vessel length density-temporal deep	0.19 ± 0.05	0.2 ± 0.05	0.63	0.2 ± 0.04	0.19 ± 0.07	0.04	0.2 ± 0.05	0.2 ± 0.04	0.60
Vessel diameter index-nasal superficial	2.08 ± 0.17	2.01 ± 0.12	0.45	2.05 ± 0.17	2.09 ± 0.14	0.68	2.06 ± 0.16	2.01 ± 0.12	0.55
Vessel diameter index-nasal deep	1.93 ± 0.14	1.9 ± 0.13	0.87	1.92 ± 0.14	1.93 ± 0.14	0.70	1.93 ± 0.13	1.88 ± 0.18	0.59
Vessel diameter index-temporal superficial	2 ± 0.17	1.93 ± 0.14	0.53	1.99 ± 0.17	1.95 ± 0.15	0.92	1.98 ± 0.16	1.94 ± 0.19	0.65
Vessel diameter index-temporal deep	1.84 ± 0.21	1.87 ± 0.17	0.62	1.85 ± 0.19	1.84 ± 0.22	0.84	1.86 ± 0.19	1.72 ± 0.2	0.97

*CI* confidence interval, *SD* standard deviation, *CAS* clinical activity score, *Log-MAR* logarithm of the minimum angle of resolution, *IOP* intraocular pressure, *MRD1* margin-reflex diameter 1, *MRD2* margin reflex diameter 2, *Hx* history.

### AS-OCTA parameters pre- and post-surgery

**Figure 1:**
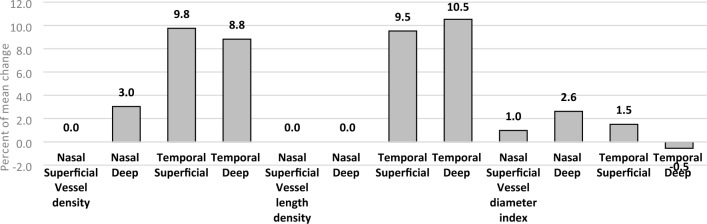
The percentage of mean changes in AS-OCTA parameters after decompression surgery.

AS-OCTA parameters were measured one week before and six weeks after decompression surgery. Analysis of these data revealed that most of the temporal vasculature measurements (vascular density (in females), vascular diameter index, and vascular length index, both in the superficial (only in females) and deep layers) decreased significantly six weeks post-surgery (p < 0.05). However, changes in the nasal region did not reach statistical significance. The complete results of the preoperative AS-OCTA measurements, including confidence intervals and p-values of the comparisons, are presented in Table 3. Figure 1 show the percentage of mean changes in AS-OCTA parameters at various locations.

**Table 3:** Summary of generalized estimation equation comparing ophthalmic examinations before and after decompression.

Characteristics		Coefficient	95% CI		P-value
VA (Log-MAR)	Male	**0.11**	**0.04**	**0.18**	** < 0.01**
	Female	0.02	− 0.03	0.07	0.45
Exophthalmos	Male	**− 4.09**	**− 4.86**	**− 3.32**	** < 0.01**
	Female	**− 2.82**	**− 3.36**	**− 2.29**	** < 0.01**
MRD1	Male	**− 0.50**	**− 0.9**	**− 0.10**	**0.01**
	Female	0.17	− 0.36	0.71	0.52
MRD2	Male	**− 0.86**	**− 1.48**	**− 0.24**	** < 0.01**
	Female	**− 1.42**	**− 1.79**	**− 1.05**	** < 0.01**
IOP	Male	− 1.73	− 4.03	0.58	0.14
	Female	**− 2.00**	**− 3.15**	**− 0.84**	** < 0.01**
Vessel density-nasal superficial	Male	− 0.014	− 0.04	0.02	0.40
	Female	− 0.021	− 0.06	0.02	0.30
Vessel density-nasal deep	Male	− 0.01	− 0.09	0.07	0.80
	Female	− 0.03	− 0.08	0.01	0.16
Vessel density-temporal superficial	Male	− 0.04	− 0.09	0.02	0.17
	Female	− **0.08**	− **0.11**	− **0.04**	** < 0.01**
Vessel density-temporal deep	Male	− 0.07	− 0.14	0.003	0.06
	Female	− **0.09**	− **0.13**	− **0.04**	** < 0.01**
Vessel length density-nasal superficial	Male	− 0.005	− 0.02	0.01	0.51
	Female	− 0.01	− 0.03	0.01	0.21
Vessel length density-nasal deep	Male	− 0.002	− 0.05	− 0.04	0.9
	Female	− 0.01	− 0.04	0.005	0.15
Vessel length density-temporal superficial	Male	− 0.02	− 0.04	0.005	0.12
	Female	− **0.02**	− **0.04**	− **0.01**	** < 0.01**
Vessel length density-temporal deep	Male	− **0.03**	− **0.06**	− ** < 0.01**	**0.04**
	Female	− **0.04**	− **0.06**	− **0.02**	** < 0.01**
Vessel diameter index-nasal superficial	Male	− 0.003	− 0.09	0.08	0.92
	Female	− 0.03	− 0.11	0.05	0.44
Vessel diameter index-nasal deep	Male	− 0.06	− 0.21	0.09	0.42
	Female	− 0.07	− 0.17	0.03	0.19
Vessel diameter index-temporal superficial	Male	− 0.04	− 0.18	0.09	0.52
	Female	− **0.12**	− **0.18**	− **0.05**	** < 0.01**
Vessel diameter index-temporal deep	Male	− **0.11**	− **0.21**	− **0.01**	**0.02**
	Female	− **0.13**	− **0.22**	− **0.043**	** < 0.01**

*CI* confidence interval, *SD* standard deviation, *CAS* clinical activity score, *Log-MAR* logarithm of the minimum angle of resolution, *IOP* intraocular pressure, *MRD1* margin-reflex diameter 1, *MRD2* margin reflex diameter 2, *Hx* history.

Significant values are given in bold.

### Ophthalmologic examinations pre- and post-surgery

Results from ophthalmologic examinations conducted one week before and six weeks after decompression surgery indicated significant improvements in all measurements except for the log MAR VA and MRD1 in females and IOP in males. The results of the generalized estimation equation comparing pre and post-ophthalmic examinations and AS-OCTA data in males and females including confidence intervals and p-values, are also presented in Table 3. Figures 1 illustrates the percentage of mean changes in AS-OCTA parameters after decompression surgery. The mean decrease in proptosis for patients who underwent one-wall decompression (medial) was 2.17 mm, while for those who underwent two-wall decompression (inferomedial) it was 3.97 mm (p < 0.05). The decreases in MRD2 for patients who underwent medial and inferomedial wall decompression were 0.79 mm and 1.5 mm, respectively (p < 0.05).

To determine which demographic variables and changes in ophthalmologic examinations significantly influenced changes in AS-OCTA parameters, we conducted a multivariate analysis, selecting these variables as cofactors. This analysis focused on the AS-OCTA parameters of the temporal region, as changes in the nasal region were not significant. The multivariate analysis revealed that sex and history of thyroidectomy had a significant modifying effect on changes in the superficial layer of the temporal half of ocular surface vascularity after decompression surgery. Additionally, changes in Hertel exophthalmometry and intraocular pressure (IOP) were significantly correlated with changes in the deep layer of the temporal half of ocular surface vascularity post-decompression. Table 4 presents the demographic data and changes in ophthalmologic examinations that significantly impacted these parameters.

**Table 4:** Summary of multivariate analysis of influencing cofactors on AS-OCTA parameters changes after decompression surgery.

Characteristics	Region	Layer depth	Co-factor	B	Std. error	P value
Temporal vessel density		Superficial	Sex	0.169	0.0246	< 0.001
			Thyroidectomy	− 0.227	0.0388	< 0.001
			CAS	0.138	0.0347	< 0.001
		Deep	Exophthalmos	0.065	0.0193	0.001
			IOP	0.018	0.0090	0.049
Temporal vessel length density		Superficial	Sex	0.049	0.0082	< 0.001
			Thyroidectomy	− 0.073	0.0116	< 0.001
			Age	0.001	0.0003	0.008
			VA (Log-MAR)	− 0.105	0.0478	0.028
			CAS	0.055	0.0104	< 0.001
		Deep	Thyroidectomy	− 0.069	0.0233	0.003
			Exophthalmos	0.027	0.0075	< 0.001
			MRD1	0.037	0.0142	0.010
			IOP	0.011	0.0042	0.010
Temporal vessel diameter index		Superficial	Sex	0.290	0.0693	< 0.001
			Thyroidectomy	− 0.374	0.0911	< 0.001
			Exophthalmos	0.074	0.0278	0.007
		Deep	Sex	0.189	0.0756	0.013
			Exophthalmos	0.058	0.0245	0.017
			IOP	0.022	0.0093	0.017

*SD* standard deviation, *IOP* intraocular pressure, *CAS* clinical activity score.

## Discussion

In this study, we evaluated changes in the superficial ocular vasculature in 31 eyes with inactive TED before and after decompression surgery using AS-OCTA imaging. Our findings showed a significant decrease in the superficial ocular vasculature in the temporal region following decompression surgery. To our knowledge, this is the first study to assess the impact of decompression surgery on the superficial ocular vasculature using AS-OCTA.

The retro-orbital crowding caused by orbital muscle and fat hypertrophy in TED decreases blood velocity in the superior ophthalmic vein (SOV) and increases intra-orbital pressure and orbital venous congestion^17–20^. This crowding can be severe enough to induce retrograde blood flow in the SOV and lead to dysthyroid optic neuropathy^21^. Venous congestion thickens the choroidal layer in patients with TED, with one study reporting an increase in choroidal vasculature from 63.34% in healthy controls to 65.9% in TED patients^15^. Additionally, orbital venous congestion raises episcleral venous pressure, which disrupts aqueous humor outflow and increases intraocular pressure (IOP)^22,23^.

This phenomenon is similar to what occurs in patients with carotid-cavernous fistula, where blood flow reversal in the SOV causes blood congestion in the episcleral venous plexus at a depth of 350–400 µm, as imaged by AS-OCTA^19^. Episcleral venous congestion elevates IOP, potentially leading to glaucomatous optic neuropathy. OCTA imaging has revealed vascular dropout in the peri-papillary region, indicating early stages of glaucomatous optic neuropathy^19^. The rise in episcleral venous pressure is a major proposed mechanism for the increase in IOP in TED, which can exacerbate pre-existing dysthyroid optic neuropathy.

Studies assessing macular and peri-papillary vasculature using OCTA have shown that vascular density in the retinal and peri-papillary regions is significantly lower in TED patients compared to healthy controls^24^. The impact of TED activity on peri-papillary vasculature is complex, with potential increases in peri-papillary blood circulation due to inflammatory mediators or decreases due to increased orbital pressure and congestion from inflamed orbital tissues^24,25^. Understanding the net change in retinal and peri-papillary vasculature in active TED requires further studies with larger sample sizes.

There is a lack of published articles specifically evaluating conjunctival and scleral vasculature changes after decompression surgery. However, at the 2018 ARVO annual meeting, Chung reported that deep episcleral venous velocity and vascular density increased post-decompression surgery in TED patients, though the sample size was small^16^. This highlights the need for more extensive research on this topic.

We did not find a significant correlation between the number of decompressed walls and changes in AS-OCTA parameters. Similarly, there was no significant correlation between Hertel exophthalmometry and changes in AS-OCTA parameters. However, multivariate analysis revealed that postoperative changes in Hertel exophthalmometry readings and intraocular pressure (IOP) measurements were significantly correlated with changes in AS-OCTA parameters in the deep layer of the ocular surface vasculature. Therefore, future studies focusing on the deep layer of the episcleral and conjunctival vasculature are necessary to better understand the pathogenesis of IOP increase in patients with TED.

As mentioned above, glaucoma and ocular hypertension are more prevalent in TED patients than in healthy individuals^22,26^. One of the primary suggested causes of this increase in IOP is elevated episcleral venous pressure^22^. Consistent with previous studies, we observed a small but statistically significant decrease in IOP following decompression surgery^27^. However, we did not find a significant correlation between the number of decompressed walls and changes in AS-OCTA parameters. Similarly, there was no significant correlation between Hertel exophthalmometry and changes in AS-OCTA parameters.

Multivariate analysis, however, revealed that postoperative changes in Hertel exophthalmometry readings and IOP measurements were significantly correlated with changes in AS-OCTA parameters in the deep layer of the ocular surface vasculature. A comparison of aqueous angiography imaging by ICG and AS-OCTA images of the deep layer of episcleral and conjunctival vasculature showed that the functional post-trabecular aqueous humor outflow patterns resemble the segmental distribution of the deep layer vasculature, which is denser in the limbal zone than in the peripheral zone^28^. This finding aligns with our results. Therefore, future studies focusing on the deep layer of the episcleral and conjunctival vasculature are necessary to better understand the pathogenesis of IOP increases in patients with TED.

A comparison of AS-OCTA parameters before and after decompression surgery revealed a significant decrease in vascular density (in females), vascular diameter index, and vascular length index in the temporal region of the ocular surface, both in the superficial (only in females) and deep layers (p value < 0.05). It appears that post-decompression surgery, the orbital pressure decreases, consequently reducing pressure on the vessels. These changes mitigate venous stasis and vascular congestion. Thus, similar decreasing changes are observed in the vasculature of the ocular surface, in addition to the vascular changes in the choroid layer. We observed a decrease in AS-OCTA parameters in the temporal region of the ocular surface following decompression surgery. AS-OCTA images were obtained one week before and six weeks after decompression surgery. Despite medial wall decompression being performed in all patients, residual inflammation in the nasal conjunctiva due to the trans-caruncular incision might have hindered a reduction in ocular surface vasculature six weeks post-surgery. A longer follow-up duration may alleviate inflammation from the surgical approach in the nasal region, providing a more accurate depiction of changes in AS-OCTA parameters in the nasal area of the ocular surface. Out of the 6 smokers in this study, 4 were men and two out of the three patients who had undergone thyroidectomy were smokers. Smoking can reduce the response to treatment for thyroid-associated ophthalmopathy. This may explain the lack of significant reduction in vascular density in men and in those who had undergone thyroidectomy. As previously mentioned, the superficial episcleral vessels radiate from the limbus, which serves as a reservoir for inflammatory cells in the ocular surface^29^. Smoking exacerbates inflammatory conditions on the ocular surface. This issue can explain the lack of significant reduction in AS-OCTA parameters in the superficial layer in men. It also provides a rationale for why thyroidectomy and gender significantly influenced changes in AS-OCTA parameters in the superficial layer in the multivariate analysis.

Other factors may contribute to the observed lack of reduction in blood flow on the nasal side of the episclera. Veins are more densely distributed on the nasal side compared to the temporal side of the episclera^30^. Following trabeculectomy, collector vessels become more prominent in the nasal periphery and episclera^31^. A study investigating the vascular anatomy of the episclera using video angiography in 15 patients failed to detect any veins on the temporal side of the episclera^32^. Consequently, it is plausible to anticipate lower venous blood flow on the temporal side, potentially leading to a greater decrease in speed of venous congestion on the temporal side compared to the nasal side.

In our study, we found that patients with TED who smoked had higher levels of proptosis (27 mm vs 24.3 mm) and MRD2 (7.9 mm vs 3.6 mm) compared to nonsmokers (p < 0.05). Recent research has shown that smoking is linked to increased CAS, decreased ocular motility, and reduced response to treatment^33–35^. However, we did not observe a statistically significant difference in the changes in AS-OCTA parameters between smokers and nonsmokers after decompression surgery.

Reducing orbital pressure and alleviating orbital and ocular venous congestion are key objectives in treating TED. Lowering venous congestion can improve ocular surface redness and decrease disease activity. Additionally, reducing orbital and intraocular pressure aids in treating and preventing compressive and glaucomatous optic neuropathy. Utilizing AS-OCTA to evaluate ocular surface vasculature should be considered a non-invasive and effective method for assessing the success of TED treatment.

## Limitation

We faced some limitations in this study. While no other study has assessed anterior segment vasculature after decompression surgery in TED patients with a larger sample size, a larger cohort might help identify more significant differences in our subgroup analyses. However, recruiting more patients who meet our strict inclusion and exclusion criteria is challenging. As our center is a referral center, patients often come from distant cities, making it difficult for some to attend longer and repeated follow-up sessions. Additionally, the ocular surface vasculature may need more time to fully respond to decompression, and postoperative inflammation may only completely subside after a more extended period. Future studies with larger sample sizes and longer follow-up times are needed to uncover more significant changes and modifying factors in ocular surface vasculature after decompression surgery.

## Conclusion

Orbital changes in thyroid eye disease (TED) lead to alterations in both the posterior and anterior segment circulation. These changes play a crucial role in the diagnosis and monitoring of TED patients. While several studies have quantitatively examined posterior segment circulation in TED, the anterior segment circulation has primarily been assessed qualitatively. AS-OCTA offers significant potential for enhancing research and clinical studies of patients with TED, providing a valuable tool for more precise evaluation and management.

## Data Availability

The data from this study are documented in Excel spreadsheets and are available upon reasonable request.
